# Plasticity of 150-Loop in Influenza Neuraminidase Explored by Hamiltonian Replica Exchange Molecular Dynamics Simulations

**DOI:** 10.1371/journal.pone.0060995

**Published:** 2013-04-10

**Authors:** Nanyu Han, Yuguang Mu

**Affiliations:** School of Biological Sciences, Nanyang Technological University, Singapore, Singapore; Oak Ridge National Laboratory, United States of America

## Abstract

Neuraminidase (NA) of influenza is a key target for antiviral inhibitors, and the 150-cavity in group-1 NA provides new insight in treating this disease. However, NA of 2009 pandemic influenza (09N1) was found lacking this cavity in a crystal structure. To address the issue of flexibility of the 150-loop, Hamiltonian replica exchange molecular dynamics simulations were performed on different groups of NAs. Free energy landscape calculated based on the volume of 150-cavity indicates that 09N1 prefers open forms of 150-loop. The turn A (residues 147–150) of the 150-loop is discovered as the most dynamical motif which induces the inter-conversion of this loop among different conformations. In the turn A, the backbone dynamic of residue 149 is highly related with the shape of 150-loop, thus can function as a marker for the conformation of 150-loop. As a contrast, the closed conformation of 150-loop is more energetically favorable in N2, one of group-2 NAs. The D147-H150 salt bridge is found having no correlation with the conformation of 150-loop. Instead the intimate salt bridge interaction between the 150 and 430 loops in N2 variant contributes the stabilizing factor for the closed form of 150-loop. The clustering analysis elaborates the structural plasticity of the loop. This enhanced sampling simulation provides more information in further structural-based drug discovery on influenza virus.

## Introduction

Influenza virus causes a great threat to people when it emerges as pandemic through reassortment during coinfection of different host species [1]. Pandemic influenza has high morbidity and mortality rates due to lack of prior immunity in humans [2], [3]. After adapted to humans, the seasonal influenza virus with high mutation rate still impacts public health [4]. In order to prevent and control the influenza virus infections, two strategies could apply: vaccines and antiviral drugs. It takes three to six months to create a vaccine in treating a newly emerged virus strain. During this period, the novel strain can spread globally, infect human and cause great damage to the economy [5]. In this lag phase, taking antiviral drugs is the only available approach in controlling and stopping influenza infections. In addition, because influenza virus infection cannot be completely prevented by vaccination, antiviral drugs are still necessary for the therapeutic treatment of influenza [6].

Neuraminidase (NA), which functions by cleaving the sialic acid on the host cells and facilitating viruses shedding, is an ideal drug target [7]. Currently, four anti-NA drugs have been approved: Oseltamivir [8], Zanamivir [9], Peramivir [10], and Laninamivir [11]. In 2006, NAs were found to be divided into two groups based on phylogenetic distinction, group-1 (N1, N4, N5, N8), group-2 (N2, N3, N6, N7, N9) [12]. Historically, Oseltamivir and Zanamivir were developed based on group-2 NA structures, which was a successful demonstration of the rational structure-based drug development strategy [13]. A recent crystal structure of a group-1 NA contains a cavity (150-cavity) adjacent to the active site which can be exploited to develop new anti-influenza drugs [12], [14]. The 150-cavity is capped by 150-loop which is composed of six residues from 147 to 152, and the sequence of 150-loop is relatively conserved in different sub-groups of influenza virus. It is noteworthy that the presence of 150-cavity is a character for group-1 NA, so that the 150-cavity in group-1 NA provides new opportunity in defeating influenza virus. Interestingly, a crystal structure of NA in 2009 pandemic H1N1 (09N1) revealed a deficient 150-cavity which is different from structures of other group-1 NAs [15]. Therefore, several questions need to be addressed: Could the new drug that specifically targeting the 150-cavity be effective on all group-1 influenza viruses? Could the 150-loop conformation of 09N1 inter-convert between the open and closed conformations? Which conformation of 150-loop is more energetically favorable in group-1 NAs? One following research discovered that the 150-loop of 09N1 prefers to exhibit in an open conformation based on normal MD study [16]. Some other works also revealed that the 150-loop conformation of group-1 NAs could exert an even wider extended 150-cavity in the simulation [17], [18], [19]. In order to extensively explore the heterogeneity of the loop conformation and provide a global free energy landscape of the 150-loop dynamics, we performed Hamiltonian replica exchange molecular dynamics (HREMD) simulations on 09N1 [15]. For comparison, one of group-2 NAs was also included in the study [20].

In this enhanced sampling method, HREMD, Hamiltonians except one replica were modified by increasing the van der Waals repulsion forces acting only within a selected group of atoms of the protein. The potential energy barriers for breaking favorable contacts within the selected atoms, such as hydrogen bonds, hydrophobic interactions, can be gradually reduced in the modified Hamiltonians. Meanwhile the majority of the interactions in the system, such as, protein-water, water-water interactions and the interaction within other part of the protein (excluding the selected group), keep intact, rendering the number of replicas needed significantly lower than that of the standard temperature REMD (TREMD) [21]. Because in TREMD, escaping from free energy local minima works through increasing temperature that influences all degrees of freedom of the system. Comparing with normal MD at room temperature, HREMD reduces the energy barrier formed within the selected group of atoms, helps the system to escape from meta-stable states quickly. Our previous research demonstrated its efficiency and accuracy in comparison with TREMD and normal MD [21], [22]. Here, HREMD simulations were performed on 09N1 NA with two different initial conformations, one being a closed 150-loop, the other being a modified open 150-cavity. For comparison, one of group-2 NAs, N2, was also included in the HREMD simulations. In all systems, Hamiltonian potentials were modified on 9 residues in NAs (residues 145–153) including the 150-loop (147–152). The free energy landscape of the 150-loop was extensively explored, and different states of 150-loop such as open, closed, intermediate conformations as well as widely-open configurations were all sampled in the simulations.

## Methods

### Simulation method

The potential of the system is composed of



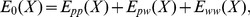
where the *E_pp_*, *E_pw_*, and *E_ww_* are the protein internal energy, the interaction energy between protein and water, and the interaction of the water molecule, respectively. In HREMD simulation, we only modify the Hamiltonian of *E_pp_(X)* term. So the *E_pp_(X)* can be further decomposed as



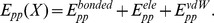




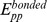
and 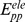 are bonded and electrostatic interaction within the protein, respective. The van der Waals terms have two components



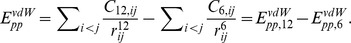



One is attractive term, 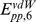, and the other is repulsive term, 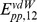. In our HREMD simulation, the repulsive term will be modified as



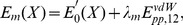
and



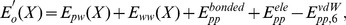
where 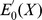 is the unmodified potential energy excluding the repulsive van der Waal interactions, and λ_m_ value is an adjustable parameter. When λ_m_ is set to 1, *E_m_(X) = E_0_(X)*. With λ_m_ value is larger than 1, the repulsive forces between atoms of protein will be enhanced. The replica exchange acceptance probability follows Metropolis criteria. Detail information can be found in Ref. 21.

### Simulation systems

Three independent systems were set up. In the first system, conformation of the NA was taken from the crystal structure 3NSS for A/California/04/2009 (09N1). In this structure the 150-loop is in the closed conformation, thus, this system is abbreviated as N1c system [15]. In order to get efficient sampling and to speed up the convergence of simulations, the same structure of 09N1 NA with an open 150-cavity was build through SWISS-MODEL sever [23] based on the structure of 2HTY for A/Vietnam/1203/04 [12], abbreviated as N1o system. Recently, a mutant 09N1 crystal structure with an open 150-cavity were published [24]. After pair wise alignment and superimposing all α carbon atoms of the modeled N1o structure onto the open-state 09N1 crystal structure, an all atom RMSD value of 0.292 Å was obtained. This small RMSD value indicates that the modeled N1o structure is very similar to the crystal structure, and provides validation of the initial open-state structure used in the HREMD simulation. One of group-2 NAs, with pdb entry 1NN2 for A/Aichi/3/67 (N2) was chosen for the third system, to compare change of loop conformation between different sub groups of NAs [20]. All systems were set up with the same procedures. The NA in each system was solvated in an octahedron box with TIP3P water [25], and the minimal distance between the protein and the edge of the box was set to 0.8 nm. Sodium and chloride ions were added with a concentration of 100 mM to neutralize the system. Protonation states for histidines were determined by Chimera software [26]. The GROMACS [27] program suite version 4.5.5 and Amber99SB force field [28] were used in all the simulations. The details of the simulation systems can be found in Table 1.

**Table 1:** Detailed information of all the MD simulation systems.

system	No. of atoms	No. of water	time (ns)	No. of replica
N1o (09N1 with open 150-loop)	35507	9889	300	6
N1c (09N1 with closed 150-loop)	35507	9889	300	6
N2 (N2 with closed 150-loop)	34630	9569	300	6

Each HREMD simulation was performed with six replicas at *T* = 300 K. The Hamiltonian potential was modified on 9 residues including the 150-loop in NAs (145–153). The values of λ_m_ were 1, 1.05, 1.11, 1.17, 1.23, and 1.3 [21]. The values of λ_m_ were chosen to achieve nearly equal exchange acceptance ratio between neighboring replicas which was averaged around 30%. All the simulations ran for 300 ns per replica in an isothermal-isobaric ensemble (300 K, 1 bar), and 5 ns normal MD equilibration simulations were performed before HREMD simulations with all heavy atom of protein restricted. Bond length constrain was applied to all bonds that contain hydrogen based on LINCS protocol [29]. Therefore, an integration step of 0.002 ps is allowed in simulations. Each replica was set to attempt exchanging at every 500 integration steps (1ps). Electrostatic interactions were treated with Particle Mesh Ewald method with a cutoff of 9 Å, grid spacing for the FFT grid <1.2 Å [30]. The cutoff 12 Å was used in the calculation of van der Waals interaction. GROMACS 4.5.5 source code was modified by our group in the exchanging step when perform detailed balance. The modified source code will be available upon request.

### Analysis method

#### Root mean squared deviation (RMSD) calculation

RMSD of 150-loop was calculated by superimposing the protein excluding the 150-loop to reference structure. Here the reference structure is the crystal NA structure with an open or closed conformation of 150-loop, and all the calculated snapshots are from *H0* trajectory, in which no modified Hamiltonian potential is applied.

#### Volume of 150-cavity calculation

The volume of 150-cavity was calculated using a python script called POVME [31]. First, a 3D-grid was built fully covering the 150-cavity of a reference open structure of NA, i.e., a crystal PDB structure with open form of 150-loop. The NA structural snapshots were extracted from *H0* trajectory every 20 ps, and superimposed onto the reference open structure. The previously generated 3D-grid was also superimposed. Those grid points near protein atoms in the structural snapshots were systematically deleted. Then the volume of the 150-cavity was calculated by a measurement script through counting the remaining grid points. The residues which influence the volume include all the residues from 147 to 152 in 150-loop as well as the nearby residues coming close to the cavity during the simulation.

#### Pair wise clustering analysis

Pair wise gromos clustering analysis (*g_cluster*) was performed, based on RMSD of 150-loop [32]. The snapshots to do the clustering analysis were selected at every 50 ps (total 6,000 snapshots in each system) from *H0* trajectory.

#### Dihedral principal components analysis (dPCA) of 150-loop

dPCA was performed to describe the conformation change of 150-loop [33]. Before dPCA, all the backbone dihedral angles of 150-loop were collected from *H0* trajectory from N1o and N1c system at every 1 ps. With these components prepared, dPCA was performed, and then the free energy landscape can be plotted. All the calculated snapshots were projected to the first two eigenvectors to get a coordinate, in this way to make a two-dimensional free energy landscape. In this analysis, data of all the backbone dihedral angles of the 150-loop in both N1o and N1c systems were dumped together, so that the projections of the dPCA data from the two systems are comparable.

#### Transition door way analysis

In order to investigate the structural transforming pathway of 150-loop, a structural evolution map was constructed based on all simulation ensembles. In the transition door way finding, structural snapshot at every 200 ps of all six trajectories (in total 18,000) were selected to perform clustering analysis with an RMSD cutoff of 0.25 nm at the first step. Then all snapshots (time interval 1ps) in six trajectories (in total 3,600,000) were compared with each cluster centers, if the RMSD between one snapshot and one cluster center is smaller than the cutoff (0.25 nm), this snapshot will be assigned to the that cluster label. Finally, the 150-loop transition door way can be traced by monitoring the cluster label changes on the time-continuous replica trajectories.

## Results and Discussion

### The configuration of 150-loop inter-converted between the open and closed conformations during HREMD simulations

**Figure 1:**
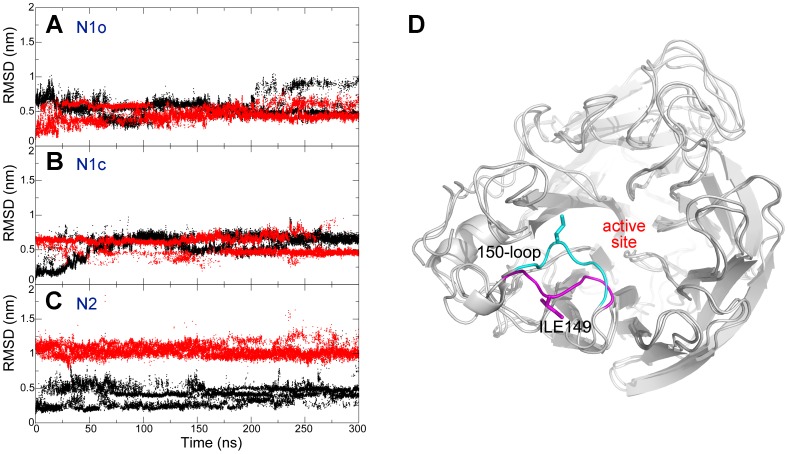
Representative NA structures together with root mean square deviation of 150-loop in three systems. Root mean square deviation (RMSD) of the 150-loop calculated by fitting the whole protein excluding the loop in N1o (A), N1c (B) and N2 (C) systems. Black and red dots represent RMSD value that calculated by fitting to N1 structure with a closed and open conformation of the 150-loop respectively. N1 structure with a closed and open conformation of 150-loop were shown in (D). Closed and open conformation of 150-loop were colored by cyan and magenta respectively.

Figure 1 shows the all atom RMSD of the 150-loop, and it indicates that this loop inter-converted between the open and the closed states many times during the HREMD simulations. In N1o system (Fig. 1A), 150-loop converted to the closed conformation with small values of RMSD with respect to the closed conformation (black dots) around 75 ns. Similar structural transforming behavior was also observed in N1c system (Fig. 1B). The RMSD values with respect to the open conformation (red dots) decreased to 0.3 nm around 25 ns, indicating a closed–to-open transition. Sometimes the RMSD values reached nearly 1 nm. It is because the RMSD was calculated by superimposing the whole protein except the 150-loop to reference crystal. Thus, the RMSD values reflect the overall movement of the loop relative to the other part of the protein. The large values of RMSD indicate that the rotational space of the loop relative to the protein is huge. In N2 system, the RMSD values with respect to the putative open conformation maintained higher than 0.75 nm indicating the sampled conformations are quite different from the putative open conformation. On the other hand, RMSD values with respect to the closed conformation mainly formed two clusters, one around 0.25 nm, and the other around 0.5 nm. This large fluctuation (the black dots) shows high heterogeneity of the 150-loop of N2.

### Volume of 150-cavity

**Figure 2:**
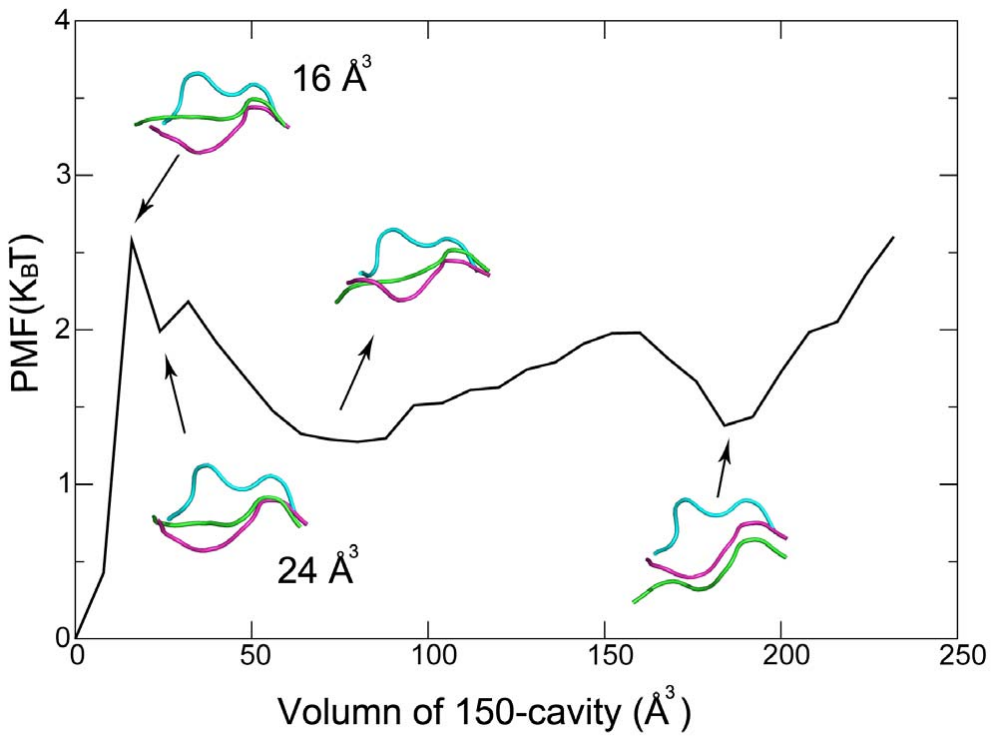
Potential of mean force (PMF) based on volume of 150-cavity. PMF calculated based on volume of 150-cavity. Structure representing two local minima together with 16 Å^3^ and 24 Å^3^ is shown as cartoon with green color. As a comparison, crystal structures with closed and open configuration of 150-loop are aligned together and colored cyan and green respectively.

The 150-cavity identified in a crystal structure of group-1 NAs was suggested to be a binding site for a new kind of antiviral drugs [12]. The dynamics of 150-loop, however, directly affects the volume of this cavity. Thus the volume of 150-cavity is also a sensitive reaction coordinate for the 150-loop conformational dynamics. The free energy profile of the system, or the potential of mean force (PMF) as a function of the volume of 150-cavity is constructed based on the simulation data with the cavity volume being calculated by POVME program (see Fig. 2) [31]. The PMF analysis was based on the ensemble trajectory in which the original Hamiltonian without any modification was applied.

To have an idea of the size of the 150-cavity, the volumes of 150-cavity of crystal structures of N2 and 09N1 were also calculated. It is 0 Å^3^ and 8 Å^3^ for N2 and 09N1 with a closed 150-loop, and 112 Å^3^ in open 150-cavity of modeled 09N1 structure. In Figure 2, two local minima with volume around 75 Å^3^ and 180 Å^3^ were identified. These two local minima with large cavity indicate that open conformation of 150-loop is favorable for 09N1. Representative structures obtained from cluster analyzing at different locations on the PMF were shown. To identify a cutoff value for determining whether the 150-cavity is open, each representative structure is checked manually. The volume size of 16 Å^3^ (also a local maximum on the PMF) is chosen as a threshold for classifying whether the 150-loop is open.

**Figure 3:**
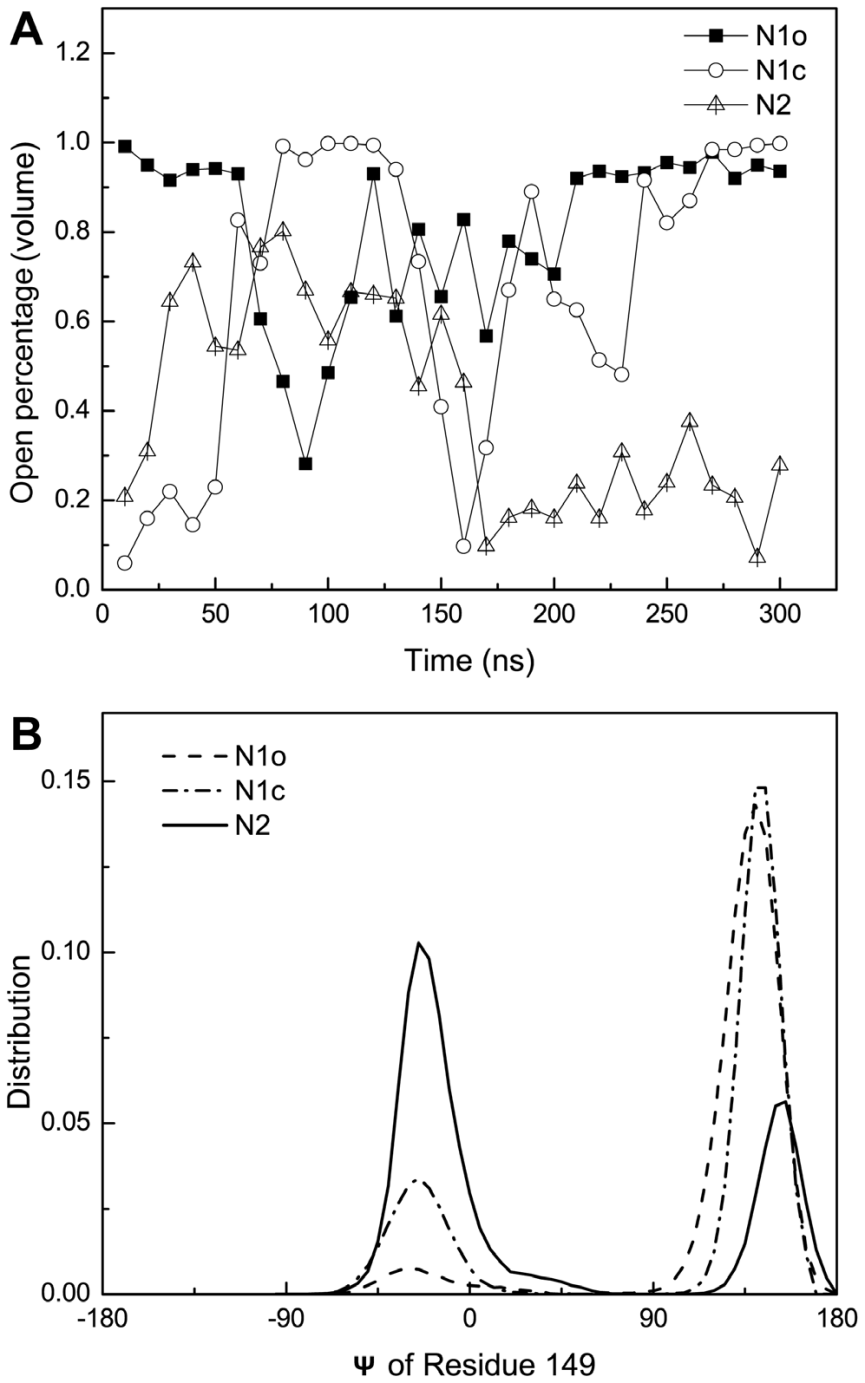
Open propensity of 150-cavity and backbone dihedral angle distribution of residue 149. Open percentage for 150-cavity is calculated based on its volume in three systems (A). Lines with square, circle and triangle represents N1o, N1c and N2 system respectively. Distribution based on psi angle of residue 149 is shown in (B). Dash, dash-dot and solid line represents N1o, N1c and N2 system respectively.

With the cutoff of 16 Å^3^ at hand, the propensity of open 150-loop can be calculated. Figure 3A shows the open percentage of the 150-cavity during HREMD simulation averaged at every 10 ns. Interestingly, both N1o and N1c systems had similarly high open propensity at the final stage of the simulations with the open percentage of 64.92% and 64.79% for N1o and N1c system (the first 50 ns simulation data were discarded). In contrast, the average open percentage of 150-cavity for the last 250 ns in N2 system is no more than 50%. Moreover, at the end stage of simulation, open propensity of N2 decreased, fluctuated around 20%. The data indicates that in 09N1 systems, 150-loop prefers to stay in a conformation with an open 150-cavity, while in the N2 system, 150-loop would rather exhibit a closed conformation.

### Backbone psi dihedral angle and side chain orientation of residue 149

The conformational change of the 150-loop is associated with the motion of backbone dihedral angles of the loop residues. After checking the backbone dihedral angles of the 150-loop residues, psi angle (Ψ) of residue I149 is found to be different between the closed and open configurations (Table S1). Ψ of I149 is −40.82° and 125.73° in the closed and open conformation of 150-loop in 09N1 structures, and this value is −29.20° in V149 of N2 crystal structure. Moreover, the side chain orientation of the residue 149 is also different between the closed and open states of the 150-loop. In the closed conformation, the side chain of residue 149 points towards the binding pocket. However, it points away from binding pocket in the open 150-loop conformation (Fig. 1D). Some work also stated that the orientation of the residue 149 are different between group-1 and group-2 NAs [12]. We found that these two variables, psi angle (Ψ) and side chain orientation of residue 149, are highly related (Fig. S1). Thus, Ψ of residue 149 can be used to characterize the different shape of the 150-loop. In Figure 3B, the distribution of the 150-loop based on Ψ of I149 in all three systems of the final 100 ns is shown. Both N1 systems evolved to mainly open states (peak with positive Ψ value). N2 system, however, demonstrates the loop favored closed conformation.

### Clustering analysis of the conformation of the 150-loop

**Figure 4:**
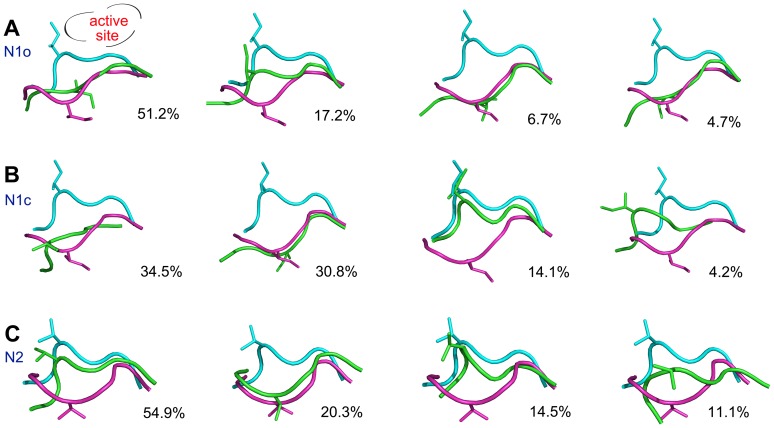
Top 4 dominant cluster center structures based on RMSD of 150-loop in three systems. Cluster analysis was based on RMSD of the 150-loop. The structures of the top 4 dominant cluster centers are shown with their cluster size in N1o (A), N1c (B) and N2 system (C) respectively. The conformation of the initial open and closed 150-loop is colored magenta and cyan respectively. The structure of each cluster center is shown in green color.

To get more details of conformations of the 150-loop in the enhanced sampling simulations, clustering analyses were carried out. In Figure 4, structures of cluster centers together with the initial structure of N1o and N1c system were plotted together. In the crystal structures, the open 150-loop usually turns away from the binding pocket and the closed loop is close to the binding pocket (Fig. 1D). For N1o system, the first, third and fourth clusters are mainly in the open form (Fig. 4A). For N1c system, the first and second clusters are marked by open loops (Fig. 4B). The proportion of open 150-loop in N1o and N1c system was 62.6% and 65.3%, and the similar values here indicate the convergence of the simulations. Conversely, in N2 system the first and third clusters, contributing 69.4% of the total ensemble, take the closed conformation (Fig. 4C). The clustering analysis discloses the detailed conformations of the 150-loop, and the resultant statistics proves that our HREMD method could provide a convergent sampling of the loop conformations within the limited simulation time.

### Turn A gauges the conformational changes of the 150-loop

**Figure 5:**
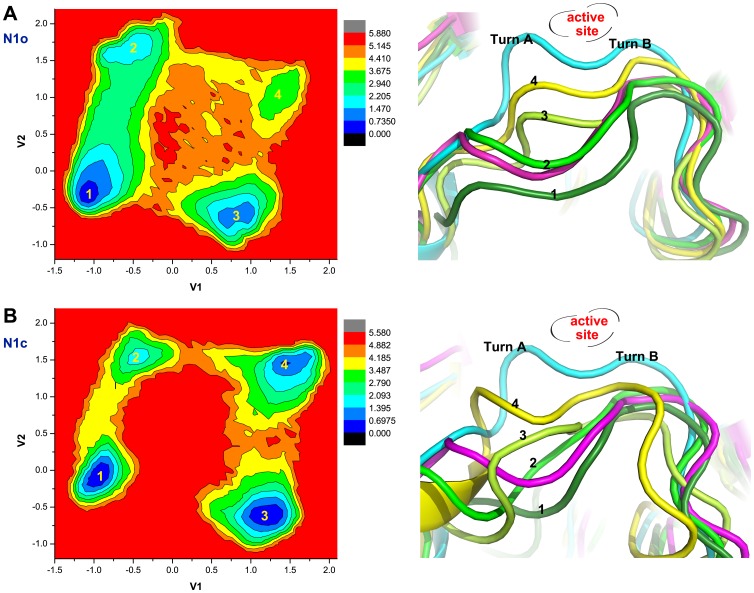
Dihedral PCA indicated turn A of 150-loop is dominant changing place. Free energy landscape obtained from dihedral PCA analysis of N1o and N1c system is shown in panel (A) and (B) respectively. Four local minima were highlight and their representative structures were shown on its right panel by clustering analysis. Structure of cluster 1, 2, 3, 4 is shown in forest green, green, lemon and yellow color. Crystal structure with closed and open 150-loop is shown in cyan and magenta color respectively.

To further characterize the conformational changes of the 150-loop, dihedral principal components analysis (dPCA) was performed [33]. In this analysis, data of all the backbone dihedral angles of the 150-loop in both N1o and N1c systems were dumped together, so that the projections of the dPCA data from the two systems are comparable. The free energy landscapes of N1o and N1c systems generated by dPCA are similar to each other (see Fig. 5). This indicates that the configuration spaces sampled in the two systems are similar, and the simulations were converged. Four local minima on the free energy landscapes were identified, and their representative structures were chosen by clustering analysis shown on the right panel. The crystal structure of the 150-loop includes two turns, and we name them as turn A (composed of residues 147–150) and turn B (composed of residues 150–152, see Fig. 5). After comparing these representative structures of local minima with the open and closed state of 150-loop in crystal structures, the position and shape of turn B are found to undergo nearly no changes. It is not the case for turn A. The 150-loop experienced the conformational transitions, from open to closed conformation, and vice verse, through altering the position and shape of turn A. Thus, turn A is identified as the structural domain to gauge the conformational transition of the 150-loop. Taken together with previous finding, the flexibility of backbone of residue 149 induces deformation of turn A, and further causes the 150-cavity to open up or to diminish. Similar results were also observed from N2 dPCA analysis (Fig. S2).

### Salt bridge of 150-loop in N2 system

**Figure 6:**
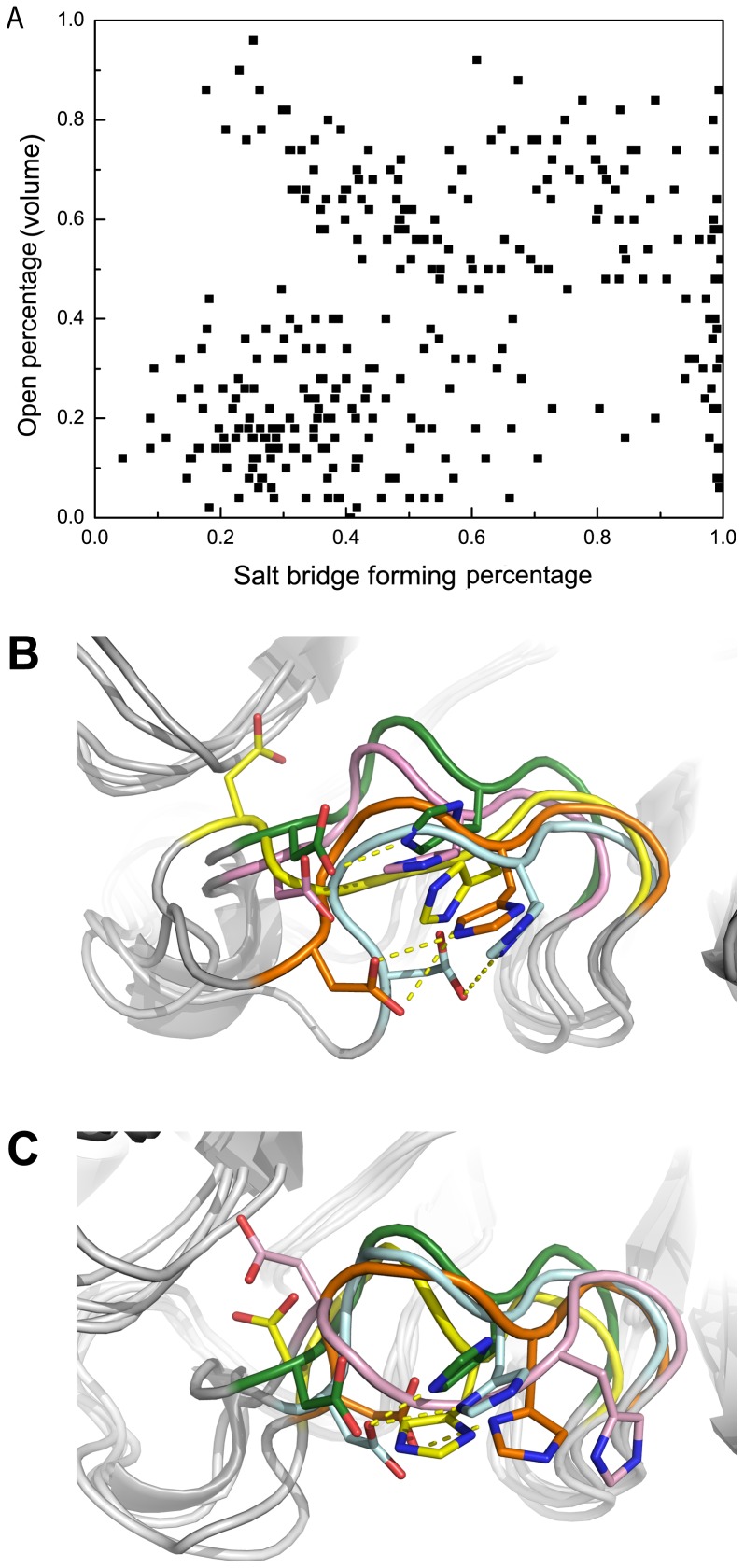
Correlation between salt bridge forming propensity and open percentage of 150-cavity. Correlation between the propensity of salt bridge forming and open percentage of the 150-loop based on volume calculation is shown in (A), both the open percentage based on volume calculation and salt bridge forming propensity were average at every 1 ns. Structures of cluster centers in the first 100 ns and the final 100 ns of N2 system are shown in panel (B) and (C). The crystal structure of N2 is shown in green color, and structure snapshots from simulations are shown in pink, yellow, orange and palecyan color.

In group-2 NAs, there is an atypical interaction of N2 with a D147-H150 salt bridge in the 150-loop. This salt bridge was believed to lock the 150-loop in the closed conformation, and to control the formation of 150-cavity [16]. With the current enhanced sampling technique applied, multiple events of breaking and formation of the salt bridge happened. Interestingly, in the HREMD simulation, it is found that the probability of salt bridge formation does not have correlation with the open propensity of the 150-loop gauged by the volume of the 150-cavity (Fig. 6A).

The structure snapshots shown in Figure 6B and 6C were obtained from the clustering analysis in the first and last 100 ns simulations. Structures in Figure 6B show that the 150-loop is in the open conformation while the salt-bridge still formed (snapshot colored by orange and light blue) and in Figure 6C the 150-loop is in the closed conformation while most of the salt-bridge are lost (snapshot colored by orange and yellow). Our finding here is at odd with the previous belief [16]. The reason may be that in the normal MD simulations, the salt bridge rarely broke resulting in a limited sampling.

**Figure 7:**
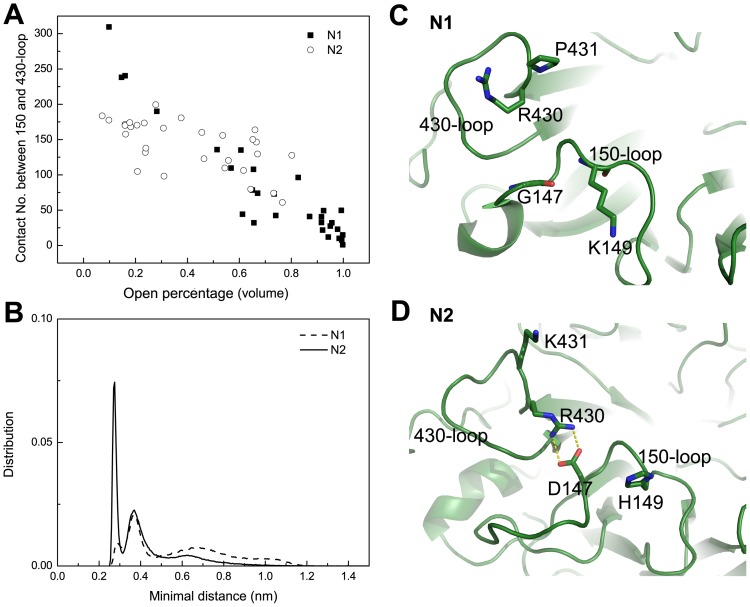
Correlation between open propensity of 150-cavity and contact number between 150 and 430 loops. Correlation between open percentage based on volume and contact number between 150 and 430-loop of two strains is shown in (A), both the open propensity of 150-cavity and contact number between 150 and 430-loop were averaged at every 10 ns. Distribution of minimal distance between heavy atoms of 150 and 430-loop is shown in (B). Panel (C) and (D) represent structure of the highest peak in N1 and N2 in (D) respectively.

Thus the reason why the N2 loop prefers a closed conformation cannot be easily explained by the presence of the salt-bridge within 150-loop. In Figure 7A, the correlation between contact number of two loops and open propensity of 150-cavity in both strains indicates that the intimate interaction between 150 and 430 loops renders 150-loop in a closed form. The dynamics of 430-loop in all three systems were investigated by calculating backbone and all atom RMSD. The backbone of 430-loop in all systems remained stable. However, the relatively larger values of all atom RMSD of 430-loop of N2 system than those of N1 systems indicated that 430-loop is more flexible in N2 system (Fig. S3). Compare the minimal distance distribution between two loops in both strains, N2 had a completely different distribution (Fig. 7B). After clustering analysis of the ensemble that contributes the highest distribution peak in N2 system, a salt bridge interaction between 150 and 430-loop was discovered (Fig. 7D). In N2 strain, when the salt bridge within 150-loop breaks, the side chain of negative charged D147 turns away and interacts with R430 in 430-loop. This intimate interaction cannot form in N1 strain (Fig. 7C) due to the sequence difference between two groups of NAs. To have a check of the conservation of the salt bridge interaction between D147 and R430 in group-2 NAs, a multiple sequence alignment analysis was performed, and the alignment result was shown in sequence logo format in Figure S4. The co-occurrence of both D147 and R430 is found with a proportion of 88.04% and 53.58% in N2 strain of homo and all species respectively. This high occurrence proportion of D147 and R430 in N2 indicates that the salt bridge interaction between D147 and R430 has a non-accidental contribution to stabilize the closed conformation of 150-loop.

### Transition doorway

**Figure 8:**
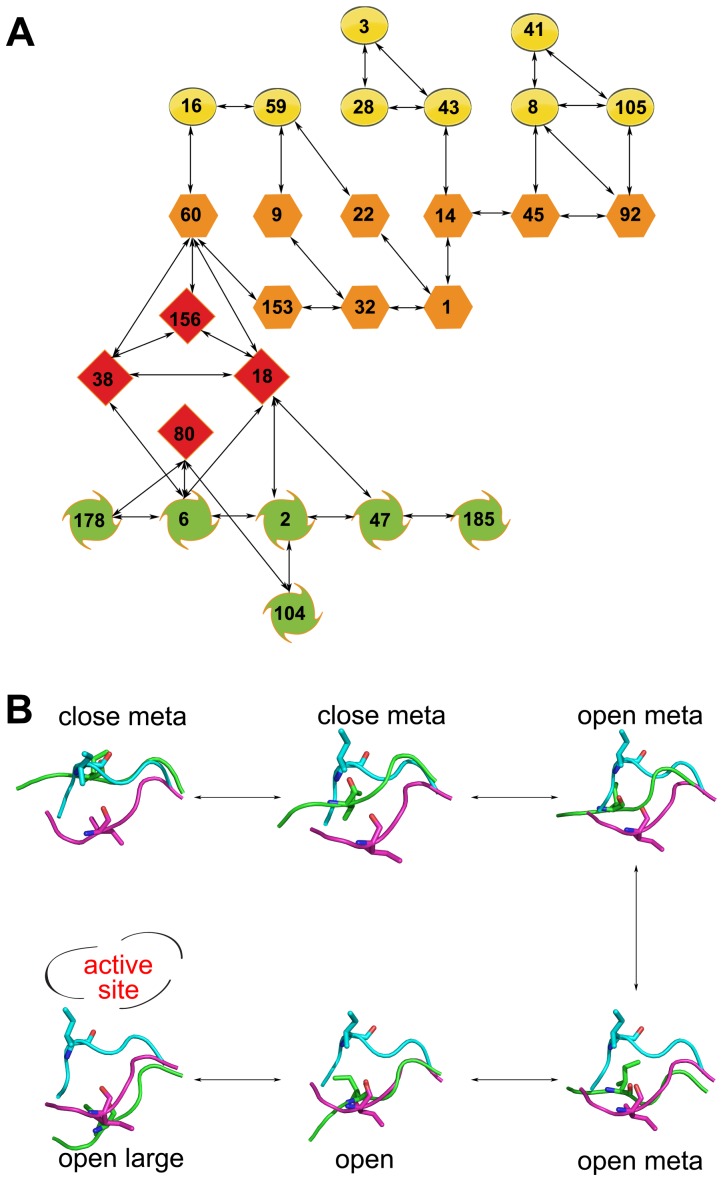
Conformational transition doorway of 150-loop in 09N1. Conformational transition doorway of 150-loop in 09N1 (A), only those with transition times larger than 100 are displayed. Symbols in yellow, orange, red and green color represent largely open, open, open-meta and close-meta conformation of 150-loop respectively; Structure snapshots with different conformation of 150-loop relating the symbols in the transition doorway (B). The initially open and closed 150-loop is colored magenta and cyan respectively. Each cluster center structure is shown in green color. ILE149 is shown as sticks.

To investigate detailed transition pathways of the 150-loop of NA in 09N1, a structure evolution map was constructed based on conformation ensemble of 09N1 systems, 3,600,000 structure snapshots in total. Firstly, structures at every 200 snapshots (in total 18,000) were selected to perform clustering analysis with an RMSD cutoff of 0.25 nm. The first largest 200 clusters which represent more than 80% of the chosen ensemble were picked out. The cluster centers were considered as the reference structures. Secondly, based on the RMSD values, all 3,600,000 structures were assigned to a member of the 200 clusters, if the RMSD of the 150-loop with respect to a certain reference structure smaller than 0.25 nm. Finally, the 150-loop transition was represented by monitoring the cluster label changes on the time-continuous replica trajectories. The transitions happened more than 100 times are shown in Figure 8. Because structures with closed conformation of the 150-loop only accounted for a small part of the simulation ensemble of 09N1 systems, this transition pathways did not catch the transition path between the closed and close-meta states of the 150-loop. In Figure 8, transition pathway of the 150-loop is shown as “close-meta” (green color)–“open-meta” (red color)–“open” (orange color)–“largely open” (yellow color) states of the 150-loop.

In Figure 8B, an example of the 150-loop conformational changing event is shown using 6 structures with different states of the 150-loop, it is noteworthy that the representative structures here are corresponded to cluster centers that plot Figure 8A. By altering the position and shape of turn A, conformation of 150-loop is changing from the closed state to the open state. This transition feature is consistent with the result obtained from dPCA analysis, which shows turn A is the dominant changing motif of the 150-loop. In addition, the side chain of I149 also changes orientation from pointing to the binding pocket (pointing upward in Fig. 8B) to other directions.

## Conclusion

In this study, a Hamiltonian replica exchange molecular dynamics method was applied to study the dynamic of the 150-loop in NAs. The crystal structure of 2009 pandemic N1 (09N1) lacks the 150-cavity. In our HREMD simulations, however, based on the PMF as a function of the volume of 150-cavity, open conformation of the 150-loop is found to be more free energy favorable in this 09N1 structure. Comparing to 09N1, the 150-loop of N2 prefers to stay in a closed configuration. We do not find evidences to support the D147–H150 salt bridge as a major factor for stabilizing the closed conformation. Instead, the more intense salt bridge interaction between the 150 and 430 loops in N2 system than in N1 accounts for the biased conformation distribution. The dPCA analysis together with transition doorway analysis discovered that the turn A (residues 147–150) of the 150-loop in 09N1 is the most dynamic motif. By altering its conformation and position, different conformation of 150-loop could inter-convert among one another. In further drug discovery studies, the potential drug molecule can be designed specifically to interact with turn A, in this way to target at one particular conformation of 150-loop. Besides, side chain orientation of I149 of NA is also an important character which highly relates with different conformation of the 150-loop.

In this HREMD sampling scheme, the modified Hamiltonians only affect the 150-loop and neighboring residues. The number of replicas needed, thus, can be as low as six. On the other hand, the other part of the protein will experience few perturbations from the modified energy functions. The sampling efficiency of that part is the same as in normal MD simulations. The comparison of residue-wise root mean square fluctuation (RMSF) between HREMD and normal MD was performed and the related discussion was added to the Support Information (Fig. S5). RMSF values of the 150-loop in HREMD are larger than those of normal MD simulation, and this phenomenon is as expected. The overall RMSF patterns are quite similar between the two data sets indicating that our HREMD scheme can selectively enhance sampling of 150-loop dynamics. If the dynamics of the 150-loop is strongly coupled with parts of protein which are located far, such as in the case of allosteric regulation [34], the sampling efficiency will be limited. Based on the data obtained, the motion of 150-loop is quite decoupled from other parts of the protein, which is an ideal paradigm for application of such HREMD method. From the methodological point of view, such HREMD method is similar to the locally enhanced sampling method [35], however, the latter usually cannot guarantee the output as a canonical ensemble.

The dynamics of the 150-loop has been found to be critical in mediating drug-protein interaction [36], [37] and drug resistance [37], [38]. Opening of the 150-cavity has become a new target for new inhibitor design [12], [14]. It is not clear what is the biological function of the plasticity of the 150-loop. As suggested by Amaro and coworkers, the opening and closing of the 150-cavity may be required for natural sialoglycan substrates to fit into the active site, given the bulky nature of these glycans [16]. Landon et al. used MD simulations and computational solvent mapping (CS-Map) method to identify that 150 and 430-loops are the novel druggable hot spots region [39]. Cheng et al. performed docking study on the crystal N1 structure as well as cluster representative structures from MD simulation, and found that half of their top hits would be neglected if only based on the crystal structure. Interestingly, these top hits have the preference to bind with the flexible 150 and 430 loops [40]. All the studies indicated the importance of identifying different conformations of NA, especially of the 150 and 430-loop region. Moreover, Cheng et al. have proposed the interaction pattern of the compound that docked into both 150 and 430-cavity. Grienke et al. have already identified a T-shaped compound that has inhibition effect and may interact with NA on an open 150-cavity [40], [41]. Our current study has made efforts in exploring in more depth the dynamic properties of the loop that provides more information for further drug discovery. This molecular insight will open new scenery to derive more potent ligands with novel scaffolds specifically targeting on the flexible 150-loop. Our group is currently pursuing in this direction.

## Supporting Information

Figure S1 **Correlation between open percentages calculated by side chain orientation of residue 149 and dihedral angle psi (Ψ) of residue 149 in three systems.** Open propensity based on psi of 149 is calculated as that, if this angle is smaller than 45° and larger than −135°, the 150-loop can be considered as closed, otherwise, it is open. Similarly, if side chain of residue 149 points towards binding pocket, it will be considered as closed, otherwise, 150-loop is open. Black, red and blue color line represents N1o, N1c and N2 system respectively.
(TIF)

Figure S2 **Dihedral PCA analysis of 150-loop in N2 system.** Free energy landscape of shown in panel A, three local minima were highlight and their representative structures were shown on its right panel (B) by clustering analysis. Structure of cluster 1, 2, 3 is shown in green, pale green and forest color. Structure with closed and open 150-loop is shown in cyan and magenta color respectively.
(TIF)

Figure S3 **Backbone and all atom RMSD of 430-loop in all three systems.** The backbone and all atom RMSD of 430-loop is shown in black and red dot respectively.
(TIF)

Figure S4 **Multiple sequences alignment and sequence logo of 150 and 430 loops for N2 strain.** Panel A and B shows the sequence alignment within homo and all species of N2 strain for influenza virus.
(TIF)

Figure S5 **The residue-wise root mean square fluctuation (RMSF) compared between HREMD N1c system and normal MD.** RMSF compared between HREMD N1c system (red curve) and normal MD (black curve). Data of normal MD came from one of our previous work [38].
(TIF)

Table S1 **Backbone dihedral angle of residues in 150-loop in 09N1 systems.**
(DOC)
