# Supplementary figures and images for: Plasticity of 150-Loop in Influenza Neuraminidase Explored by Hamiltonian Replica Exchange Molecular Dynamics Simulations
Source: PLoS One. 2013 Apr 10;8(4):e60995. doi: 10.1371/journal.pone.0060995 (PMC3622661; doi:10.1371/journal.pone.0060995)

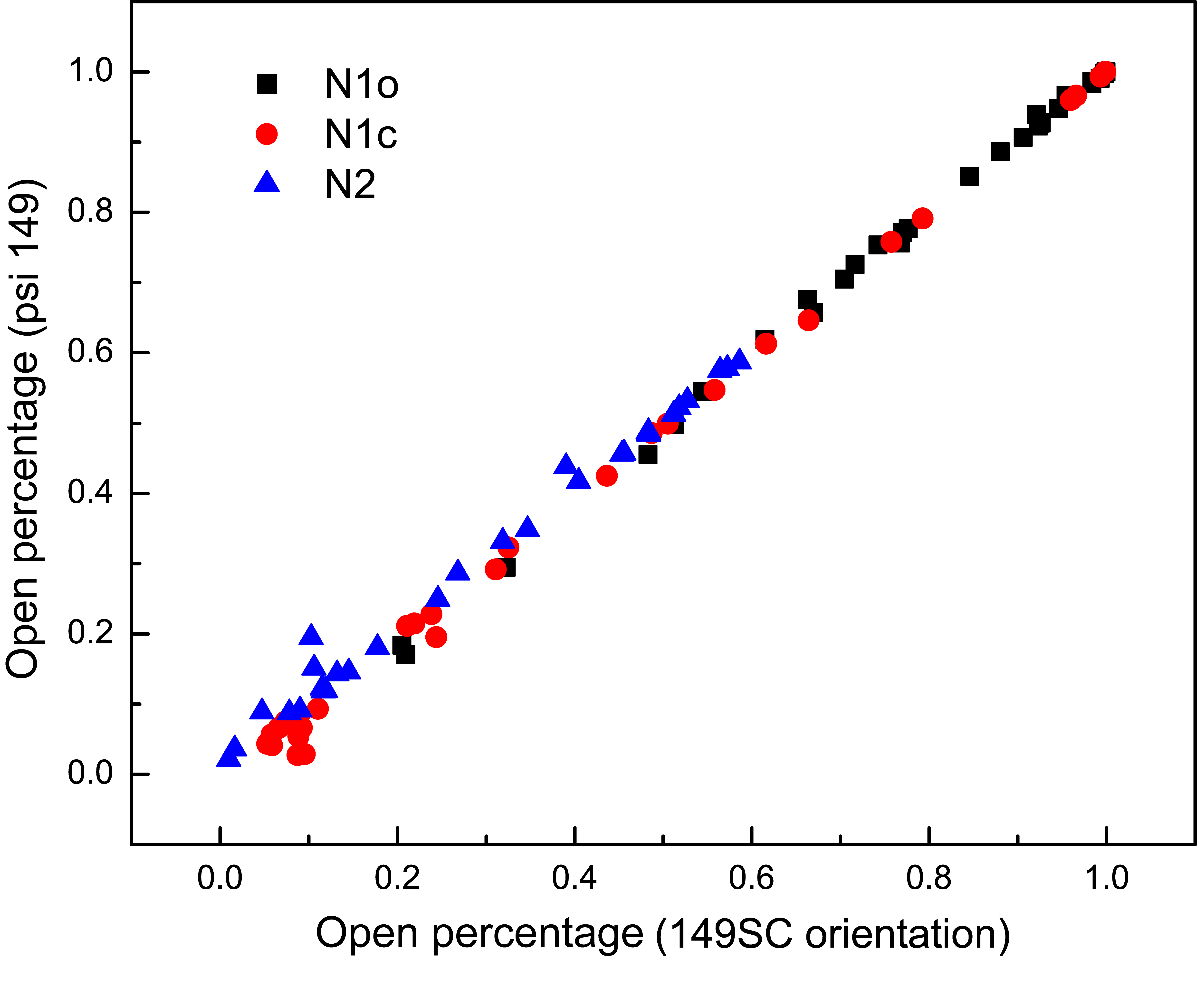

Supplement: Figure S1 — Correlation between open percentages calculated by side chain orientation of residue 149 and dihedral angle psi (Ψ) of residue 149 in three systems. Open propensity based on psi of 149 is calculated as that, if this angle is smaller than 45° and larger than −135°, the 150-loop can be considered as closed, otherwise, it is open. Similarly, if side chain of residue 149 points towards binding pocket, it will be considered as closed, otherwise, 150-loop is open. Black, red and blue color line represents N1o, N1c and N2 system respectively. (TIF) [file pone.0060995.s001.tif]

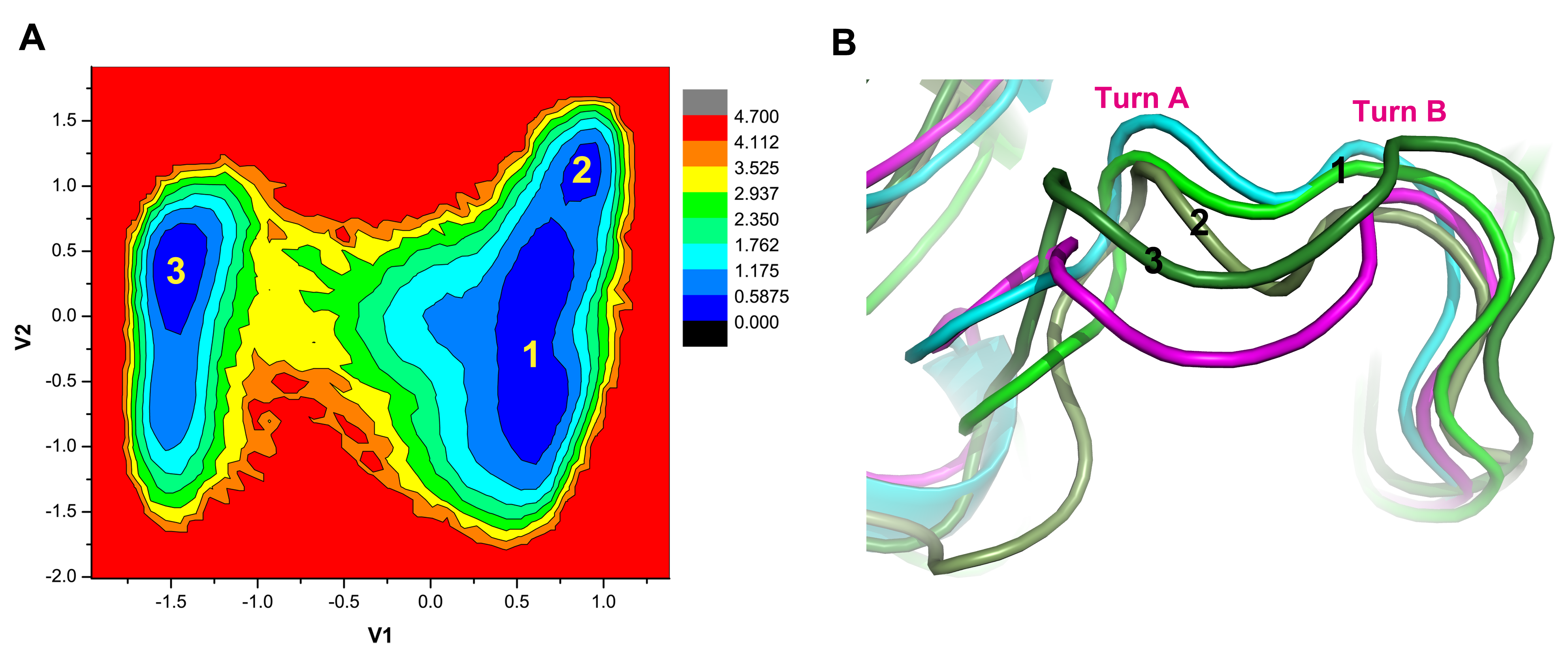

Supplement: Figure S2 — Dihedral PCA analysis of 150-loop in N2 system. Free energy landscape of shown in panel A, three local minima were highlight and their representative structures were shown on its right panel (B) by clustering analysis. Structure of cluster 1, 2, 3 is shown in green, pale green and forest color. Structure with closed and open 150-loop is shown in cyan and magenta color respectively. (TIF) [file pone.0060995.s002.tif]

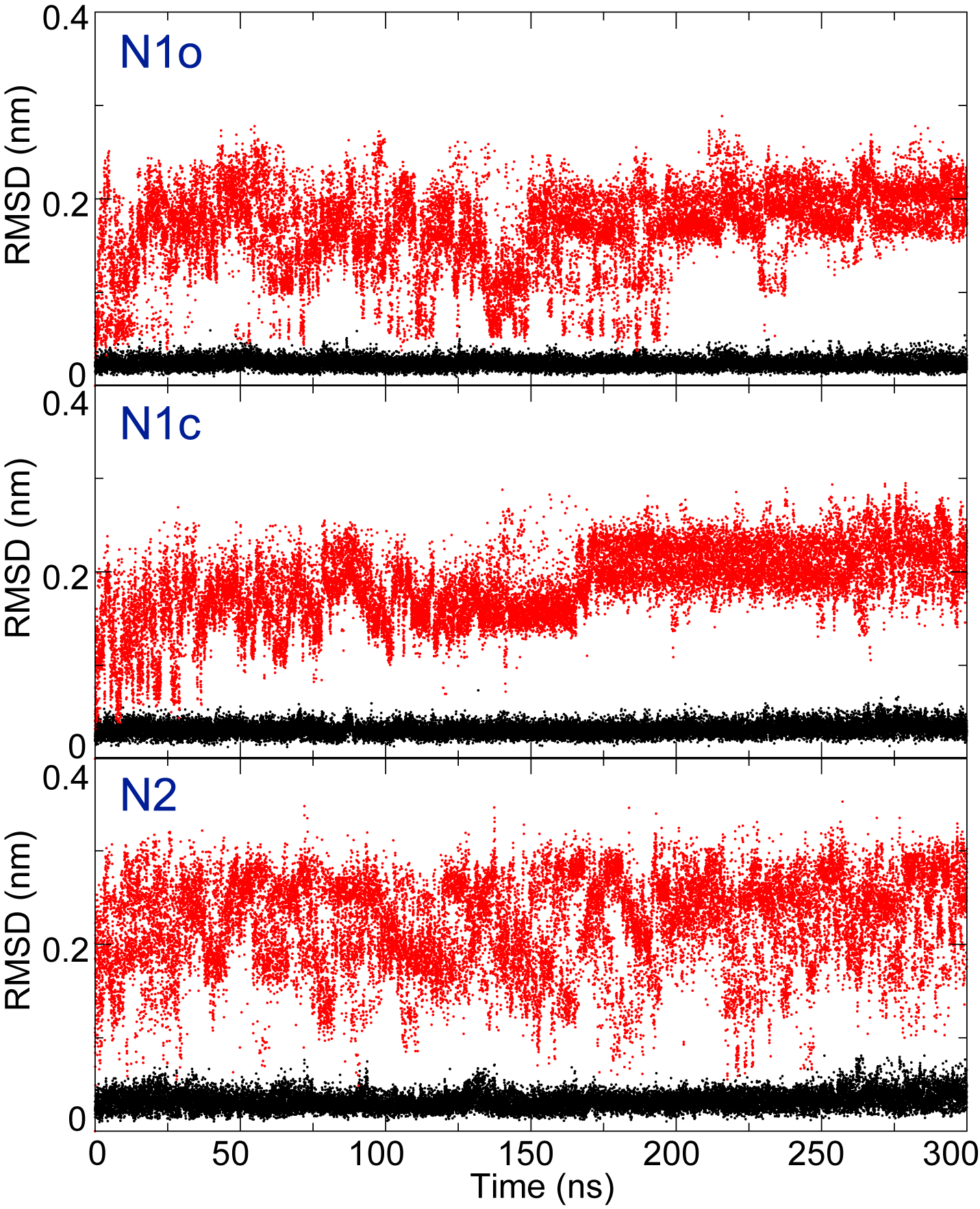

Supplement: Figure S3 — Backbone and all atom RMSD of 430-loop in all three systems. The backbone and all atom RMSD of 430-loop is shown in black and red dot respectively. (TIF) [file pone.0060995.s003.tif]

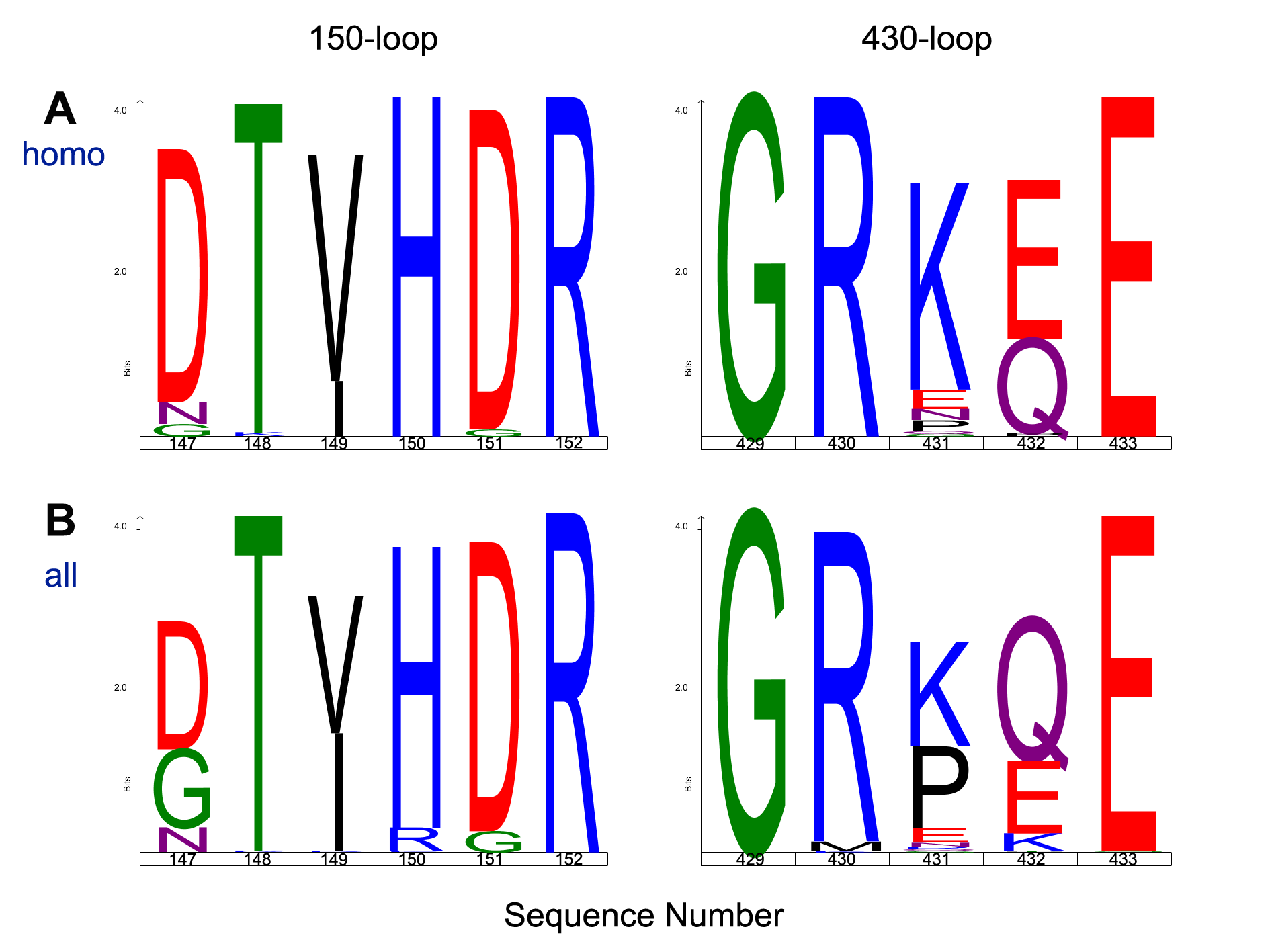

Supplement: Figure S4 — Multiple sequences alignment and sequence logo of 150 and 430 loops for N2 strain. Panel A and B shows the sequence alignment within homo and all species of N2 strain for influenza virus. (TIF) [file pone.0060995.s004.tif]

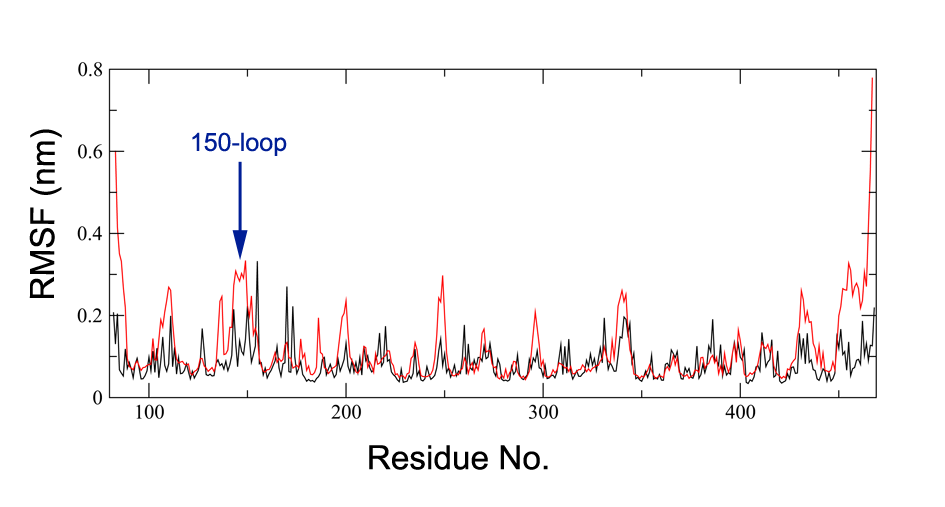

Supplement: Figure S5 — The residue-wise root mean square fluctuation (RMSF) compared between HREMD N1c system and normal MD. RMSF compared between HREMD N1c system (red curve) and normal MD (black curve). Data of normal MD came from one of our previous work [38]. (TIF) [file pone.0060995.s005.tif]
