# Supplementary material for: Plasticity of 150-Loop in Influenza Neuraminidase Explored by Hamiltonian Replica Exchange Molecular Dynamics Simulations
Source: PLoS One. 2013 Apr 10;8(4):e60995. doi: 10.1371/journal.pone.0060995 (PMC3622661; doi:10.1371/journal.pone.0060995)
Supplement: Table S1 — Backbone dihedral angle of residues in 150-loop in 09N1 systems. (DOC) [file pone.0060995.s006.doc]

**Table S1. Backbone dihedral angle of residues in 150-loop in 09N1 systems.**

| **Residues** | **Φ (N1o)** | **Φ (N1c)** | **Ψ (N1o)** | **Ψ (N1c)** |
| --- | --- | --- | --- | --- |
| **G147** | 92.40 | 79.22 | -11.50 | -2.44 |
| **T148** | -67.82 | -60.70 | -21.57 | 7.62 |
| **I149** | -65.99 | -55.35 | 125.73 | -40.82 |
| **K150** | -76.31 | -42.59 | 162.27 | 137.93 |
| **D151** | -62.48 | -76.67 | -30.90 | -47.10 |
| **R152** | -94.25 | -125.99 | 35.17 | 138.93 |
